# RETRACTION: Effects of Operating Room Nursing Intervention on Wound Infection in Patients Undergoing Ovarian Cysts Surgery: A Meta‐Analysis

**DOI:** 10.1111/iwj.70391

**Published:** 2025-03-21

## Abstract

**RETRACTION:** LinJ.‐H. , HuangW. , ZhuY.‐J. , and DuanH.‐W. , “Effects of Operating Room Nursing Intervention on Wound Infection in Patients Undergoing Ovarian Cysts Surgery: A Meta‐Analysis,” International Wound Journal 21, no. 1 (2024): e14614, 10.1111/iwj.14614.38272824 PMC10789585

The above article, published online on 15 January 2024, in Wiley Online Library (http://onlinelibrary.wiley.com/), has been retracted by agreement between the journal Editor in Chief, Professor Keith Harding; and John Wiley & Sons Ltd. Following an investigation by the publisher, all parties have concluded that this article was accepted solely on the basis of a compromised peer review process. The editors have therefore decided to retract the article. The authors did not respond to our notice regarding the retraction.

**RETRACTION:** J.‐H. Lin , W. Huang , Y.‐J. Zhu , and H.‐W. Duan , “Effects of Operating Room Nursing Intervention on Wound Infection in Patients Undergoing Ovarian Cysts Surgery: A Meta‐Analysis,” International Wound Journal 21, no. 1 (2024): e14614, 10.1111/iwj.14614.38272824 PMC10789585

The above article, published online on 15 January 2024, in Wiley Online Library (http://onlinelibrary.wiley.com/), has been retracted by agreement between the journal Editor in Chief, Professor Keith Harding; and John Wiley & Sons Ltd. Following an investigation by the publisher, all parties have concluded that this article was accepted solely on the basis of a compromised peer review process. The editors have therefore decided to retract the article. The authors did not respond to our notice regarding the retraction.

